# Computational modeling and epidemiologic approaches: a new section of the journal of translational medicine

**DOI:** 10.1186/1479-5876-10-210

**Published:** 2012-10-17

**Authors:** Michael N Liebman, Sabrina Molinaro

**Affiliations:** 1 Strategic Medicine, Inc, 231 Deepdale Drive, Kennett Square, PA 19348, USA; 2 Institute of Clinical Physiology, National Research Council, Pisa, Italy

## Abstract

A new section of the Journal of Translational Medicine is being introduced to encourage rapid communication of methods and results that utilize *computational modeling and epidemiologic approaches in translational medicine*. The focus will be on population-based studies that extend towards more molecular level analysis. Submission of studies involving methods development is encouraged where actual application and results can be shown in the healthcare and life sciences domains.

## Editorial

We introduce a new section of the Journal of Translational Medicine to encourage rapid communication of methods and results that utilize *computational modeling and epidemiologic approaches in translational medicine*. The focus will be on population-based studies that extend towards more molecular level analysis. Submission of studies involving methods development is encouraged where actual application and results can be shown in the healthcare and life sciences domains.

We acknowledge the “flood of data” emanating from new technologies, e.g. genomic sequencing, but we must also consider the enormity of data already existing in clinical histories and observational studies, nationally and internationally. There remains a critical need to re-focus translational medicine on the clinical perspective, starting with clinical need, to enhance the value of molecular-based approaches. Both perspectives fundamentally drive clinical utility and are very complementary and have been discussed in editorials in this journal [1,2].

Ultimately translational medicine must improve patient care and enhance patient-physician communication and decision-making. Patients are complex biological systems (processes) that evolve over time under the influence of many factors, e.g. environmental, lifestyle, etc. that cause an individual’s genomic risk to progress to disease. It is critical to understand, represent, analyze and interpret patients and disease, temporally, to acknowledge the complexity of disease presentation, diagnosis and treatment that go beyond genomic make-up. The successful integration of a more clinical/epidemiological base and one using high throughput technology to develop high resolution, but static, representation of a patient’s state is critical in personalized and stratified medicine.

Genomic sequencing and gene expression analysis appear yield “quantitative” data for the bioinformatics community with little experience addressing clinical or epidemiologic data. These last two are viewed as “softer” or more “qualitative” but the reality of translational medicine can only succeed these data sources approach a continuum rather than separable silos. Accurate diagnosis of a patient is required to address stratification, disease sub-typing, reliability of diagnostic criteria, physician compliance with guidelines and different EHR’s and can limit the correlation of molecular observation with clinical manifestation. Extending this correlation towards critical mechanistic understanding would become impossible. Medical informatics has focused on immediate patient management and does not necessarily address the comprehensive data collection and data models necessary to support molecular studies and analysis.

Approaches to be highlighted in this section include:

1. Data-mining for health impact: The availability of large data resources, e.g. EHR’s, PHR’s, HIE’s, registries, observational studies, etc. provide the opportunity to identify and extract patterns of patient histories, pre-disease, at time of diagnosis, response to treatment and treatment outcome that can contribute to preventive and predictive medicine and improved patient management. The development of high quality data resources and the advancement of methodologies to enable this data-mining are important aspects in making these activities translational. Additional development of appropriate ontologies to represent the complex knowledge necessary for translational research is also critical [3].

2. Data modeling: Data-modeling is both a passive and an active process. Conventional data modeling creates models to link disparate data, with a functional theme, e.g. disease progression: pre-disease to outcome. Expanding this view, to include a semantic data model, involves identifying missing data elements critical to complete a functional data model and supports the additional collection of such data [4].

3. Health record system design: The success of predictive approaches requires availability of electronic documents generated when patient and the health care infrastructure interact, including: hospital stays, surgery records, drug prescriptions, specialist health-care, death registries and EHR’s. Data flows trace the pathological history of a patient over time, and emphasize the evolution of diseases [5].

4. Applications of epidemiologic analysis: The patterns, causes, and effects of health and disease conditions in defined populations form the cornerstone of public health, and impacts policy decisions and evidence-based medicine by identifying risk factors for disease and targets for preventive medicine [6].

5. Public policy evaluation: A critical aspect of translational research is to reflect the impact of public policy as well as to potentially effect its development and implementation [7].

6. Hypothesis evaluation and testing: Robust and comprehensive analysis of the fundamental hypothesis of a study can identify early risks and opportunities and optimize study design in both clinical and observational studies [8].

7. Risk assessment in healthcare and life sciences: How early can risk be detected? How can we identify, prioritize and quantify the dimensions of risk (and opportunity?) [9].

8. Measuring and correcting for effects of missing or conflicting data: Methods to detect and potentially correct, possibly through modeling/simulation, missing or conflicting data are critical to optimize data analysis and interpretation [10].

9. Stratifying disease versus stratifying patients: Clinical observations and patient history can provide temporal portraits of disease that complement genomic identification of risk and lead to improved patient management [11].

10. Qualifying Data Content and quality: Important data sources exist containing lifestyle and socio cultural environment data, e.g. eating habits, socio-economic situation, legal substance (tobacco, alcohol and psychoactive drugs) use and illegal substance use. These data sources include surveys. Other important data may be Air Pollution Data Sources, i.e. air quality data [12].

Submitted articles should describe how the approach and results reflect the computational modeling and epidemiologic aspects of translational medicine derived both from the richness of the data available (or to be developed), and from the versatility of analysis methodologies, to contextually answer to the needs in:

prevention (identification and evaluation of modifiable risk factors)

diagnosis (optimization of early detection, stratification and staging)

therapy (stratification of disease and personalization of treatment)

health offer planning (optimization of service offerings based on volumes and health profiles of the population)

compliance (to clinical practice guidelines, to patient behavior, to following physician orders)

personalization (disease stratification based on clinical presentation)

patient management (integration of patient stratification, e.g. genomics, disease stratification and therapeutic options/outcomes)

forecasts (predictive models for patient subgroups for use in prevention, behavior modification, cost estimation, etc.)

public policy (relationship between policies and public/patient/physician response; evidence for opportunities to impact policy development)

## Competing interests

No competing interests exist.
